# Retinal OCT Parameters and Physiological Dynamics as an Effect of Anesthesia in C57BL/6J Mice

**DOI:** 10.1167/iovs.67.4.50

**Published:** 2026-04-21

**Authors:** Sybren Worm, Georg Ladurner, Lucas May, Maria Varaka, Yash Patel, Conrad Merkle, Katharina Heissl, Bernhard Baumann

**Affiliations:** 1 Center for Medical Physics and Biomedical Engineering, Medical University of Vienna, Vienna, Austria; 2 Center for Biomedical Research and Translational Surgery, Medical University of Vienna, Vienna, Austria; 3 Institute of Biomedical Physics, Medical University of Innsbruck, Innsbruck, Austria

**Keywords:** optical coherence tomography (OCT), retinal imaging, anesthesia, mouse model, retinal thickness (RT)

## Abstract

**Purpose:**

Anesthetics are commonly used to immobilize mice for ophthalmic research, however, by using anesthetics, the physiology of the mice is temporarily changed. In this work, optical coherence tomography (OCT) parameters and physiological dynamics under anesthesia are investigated for both sexes.

**Methods:**

Adult C57BL/6J mice (10 male and 10 female) were investigated in three groups. Group I was first injected with a mixture of medetomidine, midazolam, fentanyl, and ketamine (MMFK) and, after 1 week, were exposed to isoflurane and yet another week later injected with ketamine-xylazine (KX), whereas for the other groups the order of the anesthetics was permutated. Retinal OCT volumes were acquired every 5 minutes for a duration of up to a total of 60 minutes. Simultaneously, heart rate (HR), mean blood pressure (BP), and body temperature were recorded. Retinal thickness and pulsatile motions within the retina were quantitatively assessed in the OCT data and longitudinally charted. The data were evaluated through a mixed effects model and Bland-Altman plots.

**Results:**

HR, BP, and pulsatile motions within the retina differed significantly for both sexes between all anesthetics. For the retinal pulsations at the inner limiting membrane (ILM) and retinal thickness (RT), statistically significant differences were observed between male and female mice. MMFK, KX, and isoflurane also differently affected blood vessel size in the superficial vascular plexus (SVP).

**Conclusions:**

The choice of anesthesia impacts the physiology of mice. Moreover, the sex of the mouse needs to be considered in the design and comparison of longitudinal OCT investigations using methods such as OCT angiography (OCTA) or phase-based OCT modalities.

Numerous diseases are associated with the impairment or even loss of vision. In order to expand our knowledge of the pathology of ocular afflictions, studies are often performed in animal eyes providing fast-forward studies of pathologic aspects due to accelerated pathology development in the relatively shorter animal life span.^1^ Within animal experiments, mice are the most common animals available.^2^ Besides their easy handling and low space requirements, mice come with an ocular structure resembling the human eye apart from the obvious difference in size and the lack of a macula in the mouse eye.^3^ Where humans have a rod concentration of approximately 95% of all retinal photoreceptors, mice have a rod concentration of 97%.^4^^,^^5^ Moreover, models of human diseases are wildly available, including both pathologies that affect the eyes primarily and secondarily, such as glaucoma and diabetic retinopathy, respectively.^6^^,^^7^ A tool that has become a gold standard in ophthalmological diagnostics is optical coherence tomography (OCT).^8^ OCT is a noninvasive real-time imaging modality relying on low coherence interferometry, capable of reconstructing imaged volumes with micrometer scale resolution.^9^ On top of structural imaging, OCT is also vastly deployed in ophthalmic research exploiting additional contrast mechanisms such as OCT angiography (OCTA), optical coherence elastography (OCE), Doppler OCT, optoretinography (ORG), and polarization sensitive OCT (PS-OCT).^10^^,^^11^ Combining both the versatility of available mouse models of human disease with the capabilities of OCT allows for accelerated longitudinal investigations targeting specific research questions in ophthalmology.^12^^,^^13^ However, in order to effectively perform OCT imaging in mouse eyes, the mice have to be rendered immobile. This is typically done through the use of anesthetics. A commonly overlooked effect of anesthetics is that they cause temporary changes of the mouse physiology. Among other effects, this can manifest itself through a change of cardiovascular and respiratory parameters and dynamics.^14^^–^^16^ Therefore, in order to perform translational OCT research, it is critical to not only understand differences depending on mouse models or mouse sex, but also how the choice of anesthesia affects measurements and observations.

A variety of anesthesia methods have been used for preclinical research in ophthalmology.^17^ In our OCT imaging studies, three anesthetic agents have been used, which differ in administration route and mechanism: (a) isoflurane, (b) a combination of medetomidine, midazolam, fentanyl and ketamine (MMFK), and (c) a combination of ketamine and xylazine (KX). Isoflurane (ISO) is a volatile anesthetic method that quickly wears off upon removal that is commonly used within ophthalmic research.^18^ However, MMFK for mouse experiments is the anesthesia of choice recommended, for instance, by the Medical University of Vienna, and has the advantage of being partially antagonizable through the use of atipamezole and flumazenil. The mixture of MMFK is injected intraperitoneally and is based on MMF with the addition of ketamine which allows for a deeper sedation. Last, the combination of KX is an anesthetic that is injected and is commonly used for animal studies. Several studies have investigated effects of ISO, MMF, and KX in rodents,^16^^,^^19^^,^^20^ albeit no distinction between sex was made and the implications on observations made by studies using OCT are unknown. However, the vasodilative and/or vasoconstrictive effects of the used anesthesia approach change the dimensions of the blood vessels and affect pulsatile motions caused by the blood vessels and thus will likely impact structural OCT measurements (e.g., of retinal thickness [RT]), vascular mapping by OCTA, blood flow measurements by Doppler OCT, and the assessment of retinal function by ORG. For instance, ISO is known to be a vasodilator,^21^ KX consists out of both a vasodilator and a vasoconstrictor,^22^ whereas MMFK combines the effects of reported vasodilators^23^^–^^25^ and a vasoconstrictor.^22^^,^^26^ Earlier research using magnetic resonance imaging (MRI) revealed that the diameters of cerebral blood vessels in mouse models were affected differently between ISO and KX anesthetics.^27^ The cerebral and ocular vasculature share the same embryological origin and have similar physiology, suggesting related responses to anesthesia.^28^

In this work, we investigate the effects of ISO, MMFK, and KX on physiological parameters in both female and male C57BL/6J mice and quantitatively chart OCT parameters for anesthesia durations of up to 42 minutes. We hypothesize that the use of different anesthetics, through temporarily changing the physiology of the mouse, will impact OCT parameters such as RT, tissue pulsations within the retina, and vessel density observed in OCTA as well as the physiological recorded parameters, such as the heart rate (HR) and the mean blood pressure (BP). Furthermore, we hypothesize that under constant imaging conditions, sex-dependent differences will present themselves in both the physiological parameters as well as in the OCT parameters in the mice.

## Methods

### Animals and Study Protocol

**Figure 1.**
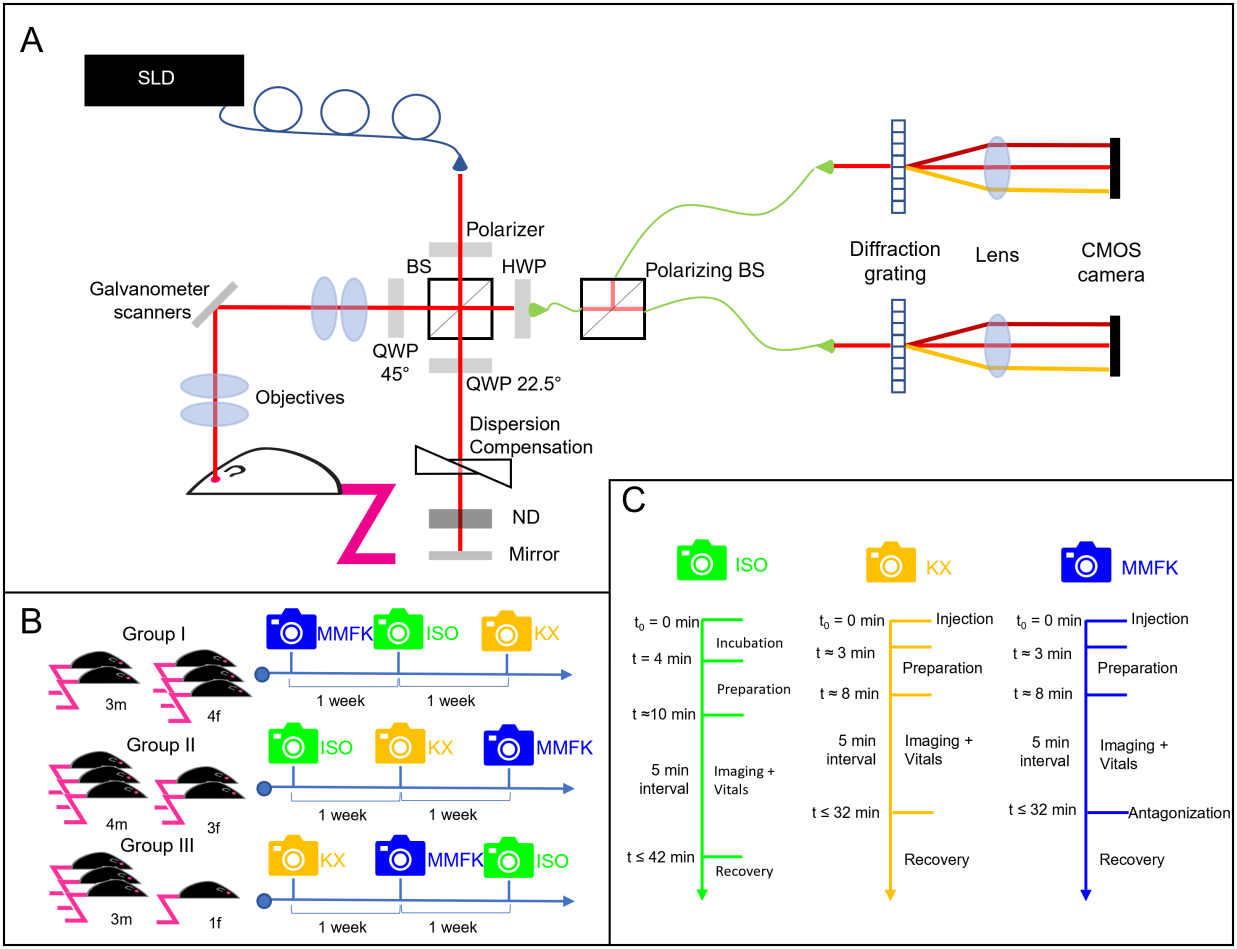
A schematic overview of the setup and imaging timeframe. (**A**) Superluminescent diode (SLD), beam splitter (BS), quarter wave plate (QWP), half wave plate (HWP), and neutral density filter (ND). (**B**) Imaging plan and composition of the groups (f = female, m = male). (**C**) Schematic overview for the imaging protocols for ISO, KX, and MMFK.

A total of 20 C57BL/6J mice aged 5 to 5.5 months (The Jackson Laboratory, strain #000664), 10 female and 10 male mice, were studied and randomly subdivided into 3 groups as visualized in Figure 1B. Group I consisted of four female and three male mice, and group II consisted of three female and four male mice, whereas group III consisted of three female and three male mice. Group I's first imaging session was performed with MMFK, the second imaging session, 1 week later, was performed using ISO, and the last imaging session, roughly a week later, was performed with KX. Similarly, group II and group III started with MMFK and KX, respectively, for the first imaging session. During the second imaging session, 1 week later, KX and MMFK, respectively, were utilized. For the last imaging session of group II and group III, MMFK and ISO were used, respectively. A schematic overview of the experimental workflow is shown in Figure 1C. During the imaging sessions, the time was logged, where *t* = 0 minute (t_0_) is denoted as the initial moment the mouse was exposed to an anesthetic. In general, starting as soon as all preparations were finished after t_0_, the mice were imaged by OCT every 5 minutes_._ For ISO, the experiment duration was generally 40 minutes, whereas for MMFK and KX, the duration was typically approximately 30 minutes. The third imaging sessions for all groups lasted an extended duration of up to 1 hour in order to investigate longer term effects. For ISO, the mice were first placed in a heated induction chamber and then exposed to 4% isoflurane in oxygen for 4 minutes. For MMFK, the mice were injected intraperitoneally with medetomidine (0.3 mg/kg body weight), midazolam (1.0 mg/kg), fentanyl (0.03 mg/kg), and ketamine (10.0 mg/kg) at t_0_ and kept warm in the induction chamber, without exposure to ISO, until they were unresponsive. Similarly, for KX, the mice were injected intraperitoneally with the mixture of ketamine (100 mg/kg) and xylazine (6.0 mg/kg) at t_0_ and kept warm in the chamber until they were unresponsive. After the induction of anesthesia, from the moment that the mice had an absence of eye motion in real time, the mice were moved to a custom-made OCT rodent imaging stage. The OCT rodent imaging stage consisted of a z-stage, a rotary z-stage, and a mouse holder that can be rotated on a right angle to the z-axis, allowing for 5 degrees of freedom to position the mice retinas in the desired location. There, the mice in the ISO cohort were exposed to 2% isoflurane in oxygen through a nose cone. For both anesthetics, tropicamide eye drops (0.5%, Agepha Pharma s.r.o., Senec, Slovakia) were used to dilate the mouse pupil. Using a noninvasive BP system (CODA Monitor; Kent Scientific, Torrington, CT, USA), the mean BP and body temperature were monitored. The mean BP was measured through arterial pulsations from the blood flow in the murine tail with the use of tail cuffs. The mean was defined as the point where the tail volume increases the most per pulsation during tail cuff deflation. The tail cuffs were fitted after the mydriatic eye drops had been administered. Finally, the body temperature was monitored throughout the imaging session through a rectal probe and the target body temperature was 38°C to be maintained through heat blankets, whereas the reached temperatures were at 37.78 ± 0.75°C. Between the OCT measurements, Oculotect eye drops (Théa Pharma GmbH, Berlin, Germany) were gently applied on the eyes and the OCT light source was turned off in order to reduce the risk of cataract induction. Before each OCT measurement, any residual eye drop was carefully removed using a cotton swab to avoid optical artifacts. After the imaging session, the mice in the MMFK cohort were injected intraperitoneally with a partial antagonist, atipamezole (1.0 mg/kg) and flumazenil (0.1 mg/kg), and were administered 1 mL glucose via subcutaneous injection to improve the recovery of the mouse. All procedures were approved by the local animal ethics committee and the Austrian authorities (protocol GZ 2024-0.044.300). All animal experiments were conducted in accordance with the ARVO Statement for the Use of Animals in Ophthalmic and Vision Research.

**Table.** An Overview of the Measured Physiological Parameters of the Mice, Through the Mean ± the Standard Deviation (std) and the Slope. The slope indicates the change of the parameter per minute as an average over all individual applicable mice slopes. The parameters were subdivided per sex for both the anesthesia methods. The RT was measured in three different configurations. The RT without alterations, the RT excluding the SVP (RT_-svp_) and the RT at the locations of the SVP (RT_@SVP_)

	Isoflurane		MMFK		KX	
Parameter	Female	Male	Female	Male	Female	Male
Heart rate
Mean ± std, bpm	495.7 ± 63.1	539.7 ± 72.2	363.7 ± 88.3	383.1 ± 90.1	309.1 ± 61.5	329.8 ± 50.6
Slope, bpm/min	2.72	3.9	0.46	0.31	0.59	1.5
Mean BP
Mean ± std, mm Hg	104.3 ± 26.7	73.6 ± 24.8	123.6 ± 19.9	111.6 ± 22.3	116.8 ± 24.1	111.7 ± 25.4
Slope, mm Hg/min	1.05	1.38	−0.34	0.11	−0.37	−0.03
MAV IS/OS
Mean ± std, nm/ms	2.36 ± 0.50	2.90 ± 1.01	2.52 ± 1.03	2.92 ± 1.02	1.82 ± 0.45	2.96 ± 0.87
Slope, nm/ms/min	0.02	0.01	0.02	0.05	−0.00	0.02
MAV ILM
Mean ± std, nm/ms	5.74 ± 0.65	6.69 ± 0.86	4.38 ± 0.99	5.58 ± 1.12	3.76 ± 0.67	5.21 ± 0.96
Slope, nm/ms/min	0.02	0.02	0.03	0.04	−0.00	0.02
RT
Mean ± std, µm	202.4 ± 7.2	199.9 ± 4.7	205.5 ± 5.8	202.5 ± 5.8	206.7 ± 3.6	198.4 ± 4.3
Slope, µm/min	−0.25	−0.09	0.05	0.00	0.12	0.04
RT_-svp_
Mean ± std, µm	201.6 ± 7.1	199.2 ± 4.7	204.8 ± 5.8	201.8 ± 4.5	206.0 ± 3.7	197.7 ± 4.2
Slope, µm/min	−0.24	−0.08	0.05	0.00	0.13	0.04
RT_@svp_
Mean ± std, µm	205.5 ± 7.7	202.7± 5.2	208.5 ± 6.2	205.2 ± 5.2	209.0 ± 3.4	201.0 ± 4.4
Slope, µm/min	−0.25	−0.12	0.05	0.02	0.12	0.03

### OCT Ophthalmoscope for Imaging the Murine Retina

The imaging during this study was performed using our PS-OCT system tailored for retinal imaging in rodents (see Fig. 1A).^29^ A superluminescent diode with a central wavelength of 840 nm and a spectral bandwidth of 100 nm served as a light source and provided an axial resolution of 3.8 µm in tissue. Two identical spectrometers were utilized to measure the interference spectra for co- and cross-polarised light. Both of the spectrometers used 3072 pixels on the line scan cameras to operate at an A-scan rate of 80 kilohertz (kHz). The sample arm optics enabled non-contact imaging with a field of view of 30 degrees × 30 degrees corresponding to approximately 1 mm × 1 mm in the mouse retina. Data sets consisting of 400 B-scans repeated 5 times for each B-scan, with each B-scan consisting of 512 A-scans, were acquired centered at the optical nerve head (ONH).

### Data Processing

**Figure 2.**
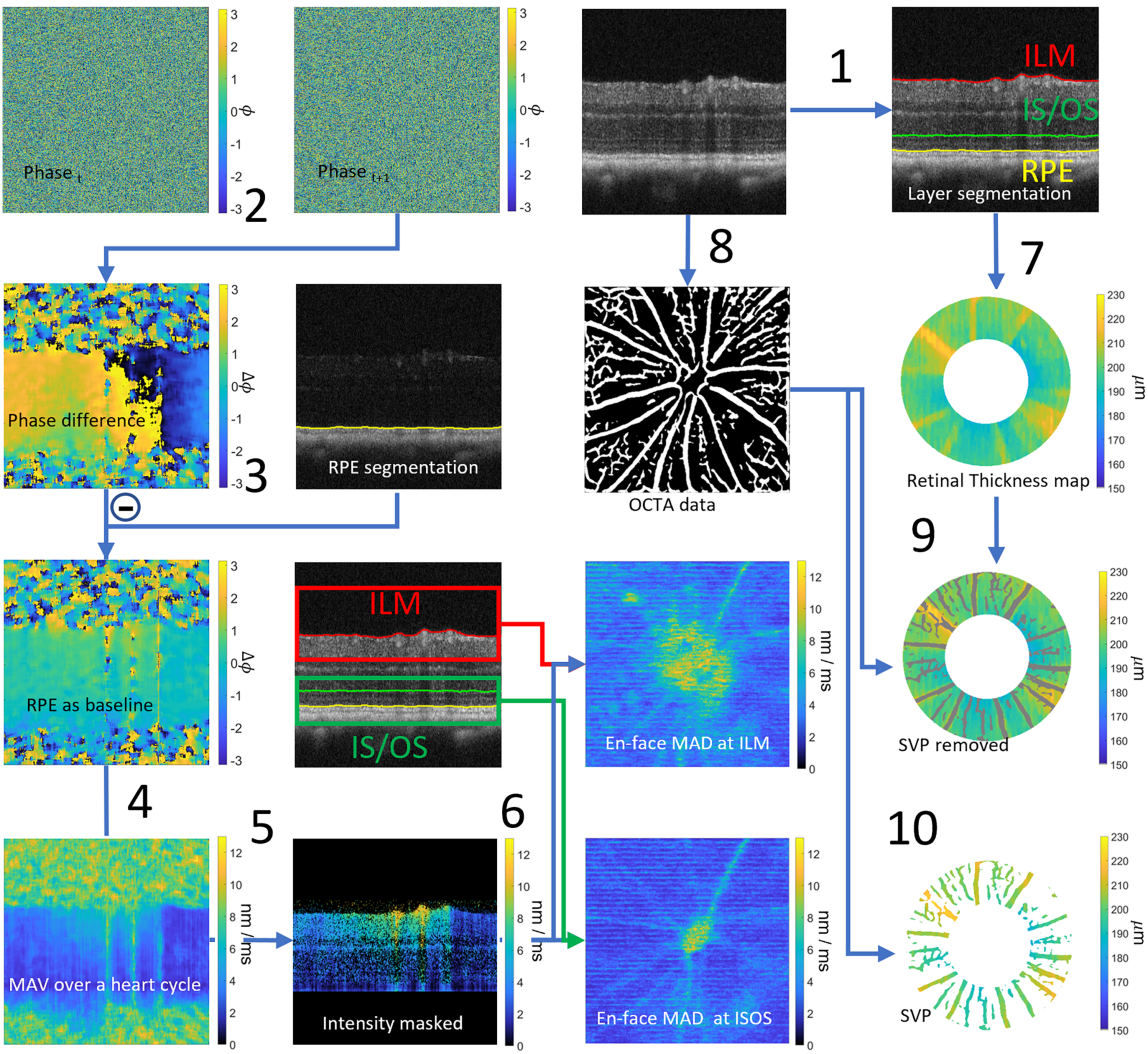
Schematic overview of the OCT data processing pipeline. (**1**) Segment ILM, IS/OS, and the RPE. (**2**) Calculate phase difference between successive frames. (**3**) Segment RPE using cross-polarized channel and use as baseline in the phase difference data. (**4**) Convert phase difference to displacement and take the mean absolute velocity (MAV) over one heart cycle. (**5**) Use intensity data to mask image and only display structures with sufficient signal to noise. (**6**) Map en face images of the MAV at the ILM and IS/OS. (**7**) Retrieve retinal thickness in an annulus around the ONH based on the layer segmentation. (**8**) Perform complex angiographic analysis to locate the SVP. (**9**) Retrieve retinal thickness without SVP. (**10**) The retinal thickness of just locations with blood vessels in the SVP segmentation.

**Figure 3.**
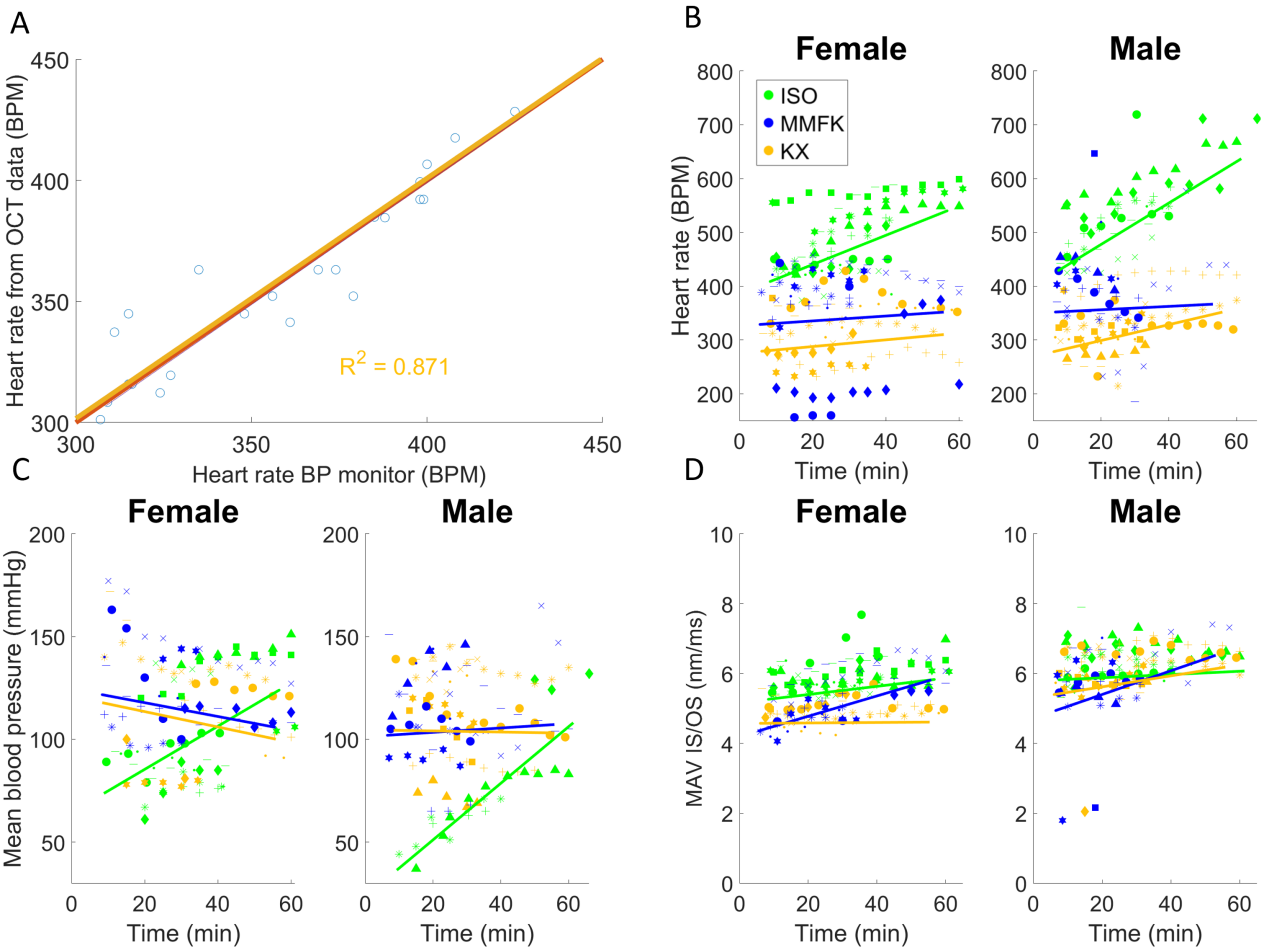
Longitudinal measurement of physiological parameters and retinal pulsation. (**A**) Proof of concept of retrieving the heart rate (HR) from the OCT data plotted against the HR measured by BP monitor, where the *red line* is the identity function and the *yellow line* is the fitted function. (**B**) HR, from OCT data, over time for female and male mice color-coded for ISO MMFK and KX in beats per minute (BPM). (**C**) Mean blood pressure over time for female and male mice color-coded for ISO, MMFK, and KX. (**D**) MAV at the position of the IS/OS over time for female and male mice color-coded for ISO, MMFK, and KX.

**Figure 4.**
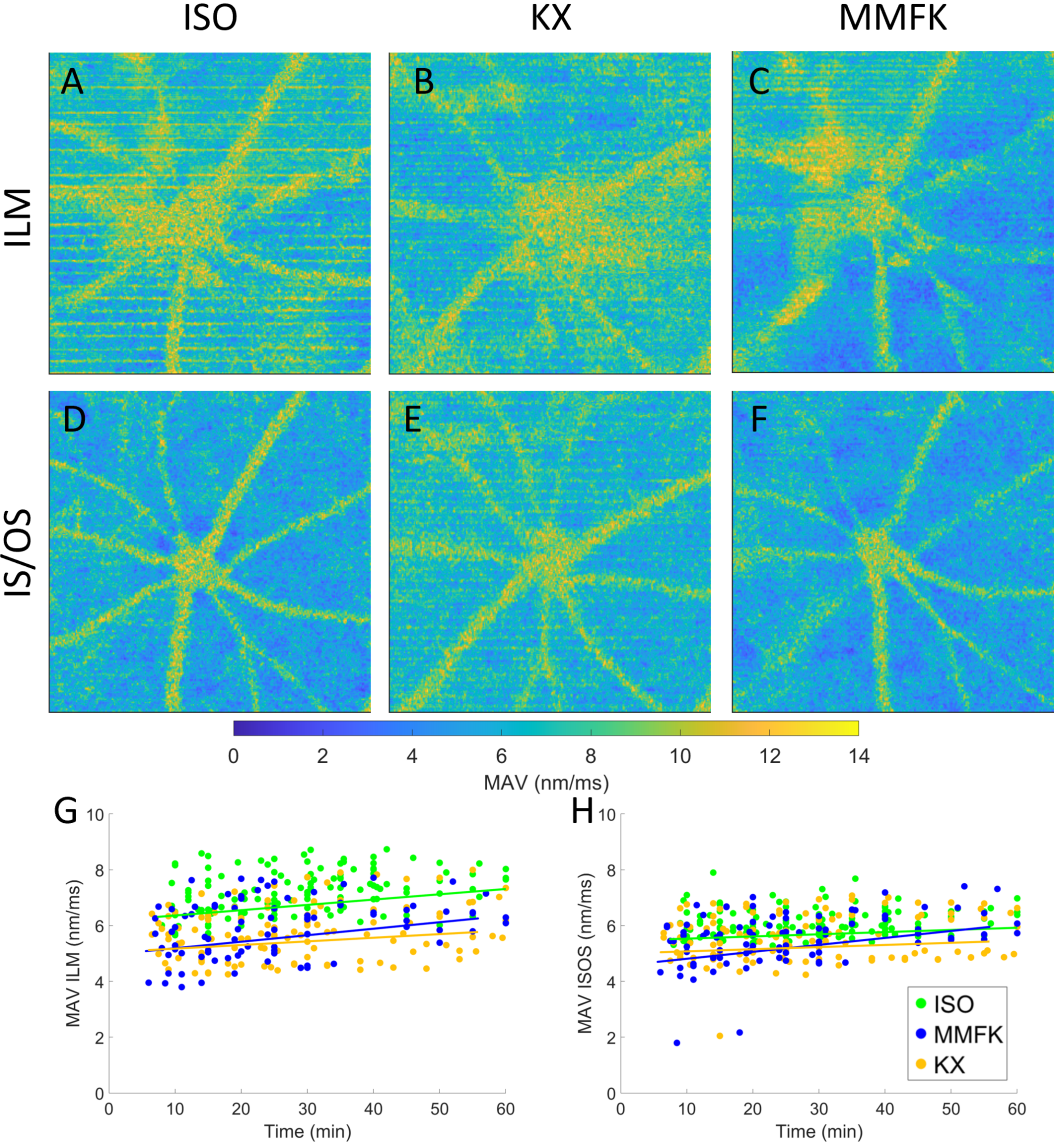
Longitudinal measurement of mean absolute velocity dynamics at the ILM and IS/OS. (**A**) Representative MAV en face maps at the ILM for ISO. (**B**) Representative MAV en face map at the ILM for MMFK. (**C**) Representative MAV en face map at the ILM for KX. (**D**) Representative MAV en face map at the IS/OS for ISO. (**E**) Representative MAV en face map at the IS/OS for MMFK. (**F**) Representative MAV en face map at the IS/OS for KX. (**G**) MAV time traces measured at the ILM for all mice, color-coded for ISO (*green*), MMFK (*blue*), and KX (*yellow*). (**G**) MAV time traces measured at the IS/OS for all mice.

**Figure 5.**
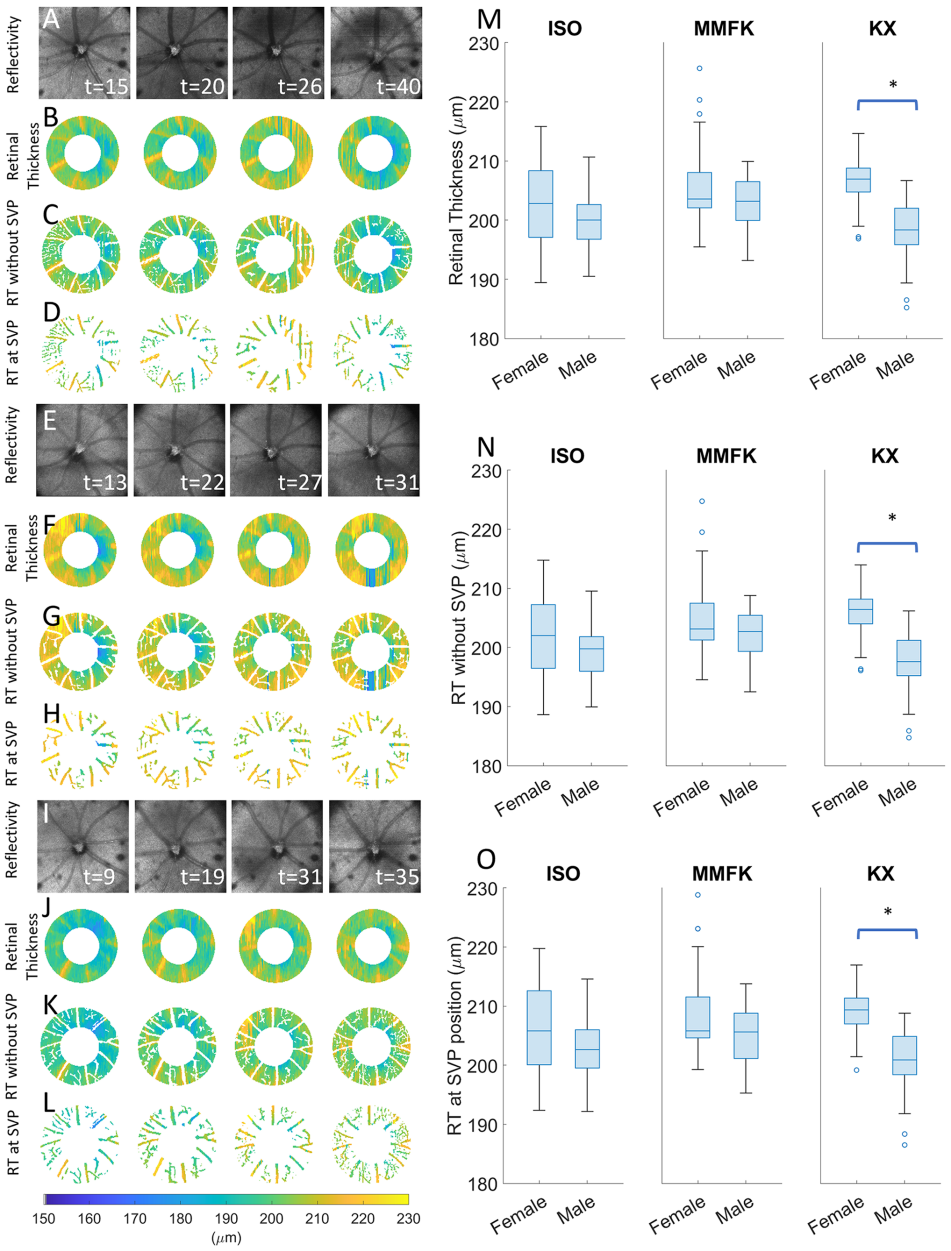
Effect of the SVP on total retinal thickness measurements for ISO, MMFK, and KX anesthesia. (**A**) Exemplary reflectivity fundus maps of a male mouse at different time points (minutes) for ISO. (**B**) Retinal thickness maps for this mouse at the various time points. (**C**) Retinal thickness maps with masked SVP. (**D**) RT maps at the locations of the SVP. (**E**) En face reflectivity fundus maps of the same mouse at different time points for MMFK. (**F**) Corresponding retinal thickness maps. (**G**) Retinal thickness maps with the SVP masked. (**H**) RT maps at the position of the SVP. (**I**) Exemplary reflectivity fundus maps of a male mouse at different time points for KX. (**J**) Retinal thickness maps for this mouse at various time points. (**K**) Retinal thickness maps with the SVP masked. (**L**) RT maps at the locations of the SVP. (**M**) Boxplot of the retinal thickness for all three anesthesia methods, subdivided into female and male, including the SVP area for the final retinal thickness. (**N**) Boxplot of the mean retinal thickness, omitting the area of the location of the SVP vessels, for all three anesthesia approaches, subdivided into female and male. (**O**) Boxplot of the retinal thickness at the position of the SVP for both anesthesia, subdivided into female and male, showing solely the mean RT of the SVP.

**Figure 6.**
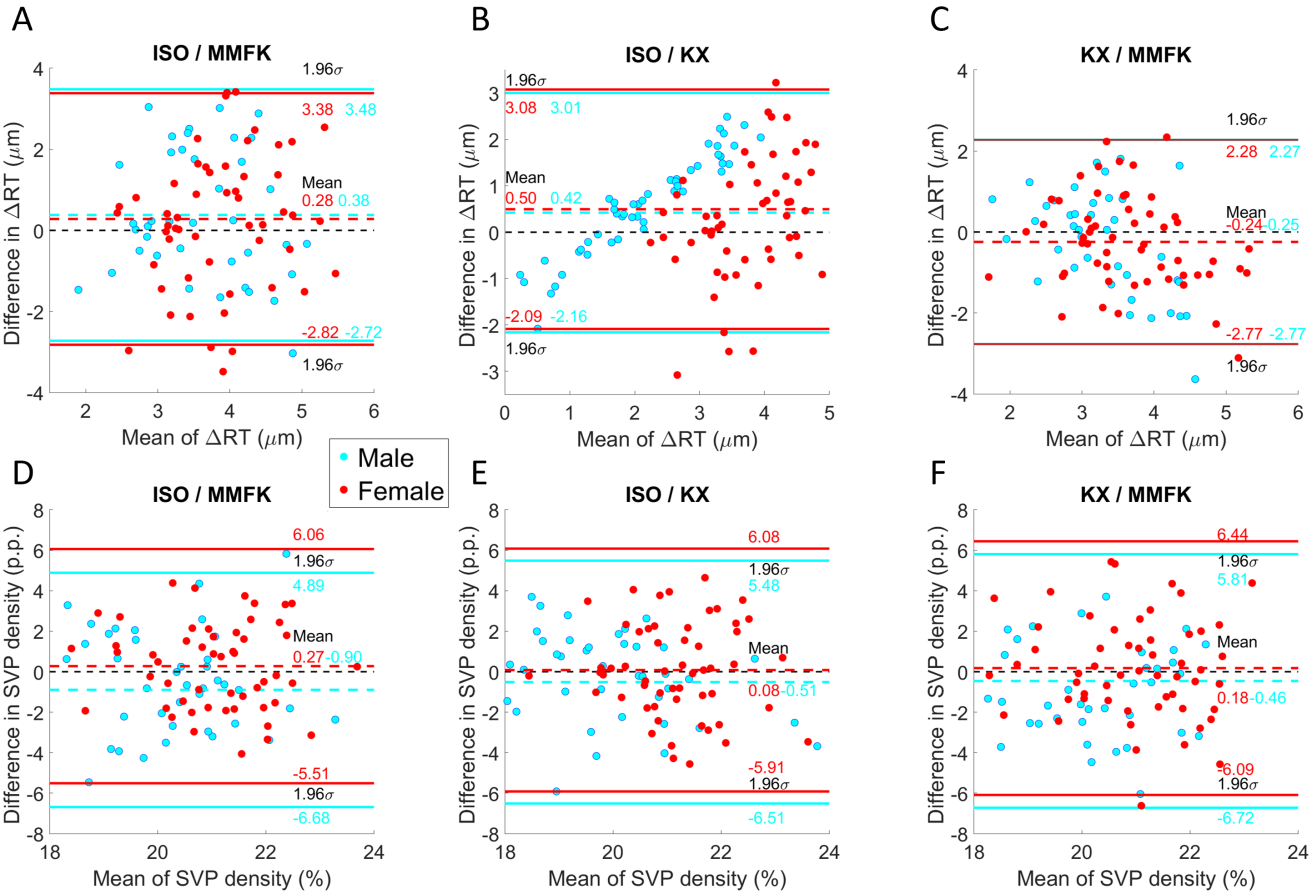
Bland-Altman plots displaying the differences in ΔRT and SVP density. The numbers and points in *red* and *cyan* correspond to female and male mice, respectively. (**A**) The ΔRT for ISO and MMFK. (**B**) ΔRT for ISO and KX. (**C**) The ΔRT for KX and MMFK. (**D**) SVP density for ISO and MMFK. (**E**) SVP density for ISO and KX. (**F**) SVP density for KX and MMFK.

The spectral raw data collected from the spectrometers were processed automatically to obtain complex-valued OCT image data, as described in our previous work.^30^ To segment the inner limiting membrane (ILM), inner-outer segment junction (IS/OS), and the upper surface of the retinal pigment epithelium (RPE) in the intensity OCT images, a custom algorithm were implemented in MATLAB R2024b. The segmentation for the ILM and RPE was based on edge detection in the co-polarized channel and cross-polarized channel, respectively (Fig. 2, 1).^31^ The IS/OS was segmented by detecting the location of intensity maxima in a slab of 15 pixels starting directly above the RPE. In addition to structural landmarks in the retina, also pulsatile deformations within the retina were investigated. Using the phase information of the OCT data, it is possible to analyze nanometer scale motions.^32^^,^^33^ Using a similar approach as described previously,^33^ first, the phase differences between corresponding pixels of consecutive B-scans were computed, and complex phasor smoothing was applied with a 10 × 20 pixel kernel (Fig. 2, 2). Second, the phase difference at the RPE was taken as a reference baseline to compensate for bulk motion, resulting in a tomogram showing the phase difference within the tissue relative to the RPE (Fig. 2, 3). From these relative phase shift data, the displacement observed between two frames was calculated for each image pixel as, $\Delta d=\frac{\lambda_{0}\Delta \Phi }{4\pi n}$ where λ_0_ is the central wavelength of the OCT light source, ΔΦ the relative phase difference, and n the refractive index of the retina, with an estimated value of 1.35 in literature.^34^ The HR was derived from these OCT displacement image data by performing a Fourier analysis of the evolution of the average ΔΦ at the position of the ILM, for all B-scans. Finally, the absolute values of the displacement data were averaged over one heart cycle, resulting in the mean absolute velocity (MAV), which serves as a parameter indicating the amount of intraretinal motion as shown in Figures 2, 4. In order to mask image regions with low intensity, which produce noisy MAV data, the intensity image was truncated below the RPE and from 10 pixels above the ILM and thresholded at 95% of the mean intensity of the B-scan and corresponding low intensity pixels were masked in black (Fig. 2, 5). To quantitatively assess pulsatile motion for different layers within the retina, the MAV was analyzed at the positions of the ILM and the IS/OS, respectively (Fig. 2, 6). To determine the RT, an annular area centered at the ONH was evaluated similar to our earlier work, where the inner circle radius was set at 0.2 mm and the outer circle radius was set at 0.4 mm.^35^ RT was computed as the axial distance between the segmented ILM and RPE layers, resulting in en face RT maps. Both the RT maps as well as the MAV maps were cropped to an annular area, where the RT is shown in Figure 2, 7 and the average RT within the annulus was calculated. The analysis of vessel density in the superficial vascular plexus (SVP) was performed through a complex OCTA analysis method described earlier.^35^^,^^36^ The threshold for SVP segmentation was set to 20 times stronger than the noise floor. An example for a mask showing the segmented vessel network in the SVP can be seen in Figure 2, 8. Finally, in order to map the effect of RT changes without the inclusion of (anesthesia-induced) diameter changes of retinal vasculature, the RT was also calculated including only pixels without vessels segmented in the SVP. Excluding the blood vessels visible in the SVP allowed for analysis of the contribution of the blood vessels to the RT (Fig. 2, 9). In the last image (Fig. 2, 10), the RT for the SVP is shown. This was used to investigate the influence of the SVP on the RT.

### Statistics

Regression analysis was performed to assess how the longitudinal data developed over time as well as to compare the HR retrieved from the OCT data to the CODA readout. For these data sets, a linear regression curve was fitted using the MATLAB 2024b curve fitter app. In order to evaluate the fit, the coefficient of determination, *R*^2^, was used. The Bland-Altman method was applied to evaluate vessel density differences caused by the three anesthesia methods, agreement of RT, and to assess the HR-measurement techniques. To statistically test for differences between the assessed parameters, a mixed effects model with a Kenward-Roger / Satterthwaite adjustments and a local Bonferroni correction were applied to the measurements of the HRs, mean arterial blood pressure, RTs, and mean absolute velocities. The fixed effects were the anesthesia, sex, and time, including all interactions. Each mouse was modeled with a random intercept to take repeated measurements into account. The models were estimated using the maximum likelihood method, and for all analyses the models converged successfully. A two-tailed paired *t*-test was used to compare differences of average RT. For all statistical tests, the level of significance was set to a value of *P* = 0.05.

## Results

### Physiological Parameters

Physiological parameters were assessed using the BP monitor and OCT. In order to validate the OCT HR data, this was plotted against corresponding HR measurements obtained from the BP monitoring device. Figure 3A shows good agreement between HR measured by the tail cuff BP monitor and the data extracted from pulsatile nano-motion in the retinal OCT data sets. The yellow line fits the measured data with an *R*^2^ of 0.871 and overlaps well with the red line indicating the identity function. Further, the Bland-Altman plot provided in Supplementary Figure S1 shows very good agreement of the HR measurement approaches.

In Figure 3B, the HR measurements over time are charted for all mice separated by female and male mice, the data are color-coded based on the anesthetic. The green data points are referring to imaging sessions performed with ISO, whereas blue indicates imaging sessions with MMFK and the yellow data points indicate imaging sessions with KX. A statistically significant difference was found for the difference in HR for female mice anesthetized with ISO compared to MMFK (*P* < 0.001) as well as KX (*P* < 0.001). For male mice, a statistically significant difference was observed for the difference in HR comparing ISO with MMFK (*P* < 0.001), MMFK with KX (*P* < 0.05), and ISO with KX (*P* < 0.001). Figure 3C shows the mean BP over time for the anesthetized mice. Again, the longitudinal data are plotted for both male and female mice and color-coded for ISO, MMFK, and KX, respectively. The mean BP for MMFK was observed to be higher for both sexes and statistically significant differences were found for both sexes compared to ISO (*P* < 0.05). Figure 3D shows the MAV over time at the IS/OS, again grouped and color-coded based on sex and anesthetic method, respectively. Table shows more details with the absolute numbers of the parameters. Supplementary Tables S1 and S2 present statistical information for the interactions of the fitted models.

### Pulsatile Retinal Motion

The MAV is a parameter representing pulsatile displacement motion within the retina with reference to the RPE. To visualize the strength of pulsatile motions in different parts of the retina, the MAV was investigated at the position of the IS/OS as well as at the ILM, thereby providing measurements both at a point close to the reference point (i.e. in the photoreceptor layer) and at the other end of the retina (i.e. the vitreoretinal interface). In Figure 4A, an en face MAV image located at the ILM is shown for a subject under ISO anesthesia. Figure 4B shows an en face at the ILM for the same mouse but for MMFK anesthesia. Similar to Figure 4A, the higher MAV can be observed around the ONH. In Figure 4C, the MAV image is depicted for the same mouse anesthetized with KX. In Figure 4D, the MAV is depicted for ISO at the IS/OS. Similar to the blood vessel locations in the ILM map shown in Figure 4A, pulsation in the blood vessels produces higher MAV compared with less vascularized parts of the retina.

At the position of the ILM, an on average approximately 2 times higher MAV can be observed compared to the IS/OS shown in Figure 4C. Higher MAV values can be observed mainly around the ONH and blood vessels. In Figures 4E and 4F, the MAV at the IS/OS is shown for anesthesia MMFK and KX, respectively. However, in the case for MMFK, the blood vessels are less clearly outlined. Time traces of MAV during the longitudinal experiment are plotted in a pooled fashion at the ILM and IS/OS in Figure 4G and Figure 4H, respectively. When assessed at the ILM, the MAV was higher for ISO measurements relatively to the MMFK and KX measurements, whereas the MAV characteristics were largely similar for all anesthesia methods at the IS/OS. MAV for ISO at the ILM was on average 31% higher than MMFK and KX. Considering that the RPE is relatively close to the IS/OS, Figure 4E and Figure 4F also show that a relatively large portion of the total MAV at the position of the ILM – namely 48.7% averaged over all MAV maps and anesthesia approaches – was contributed by the tissue motion between the RPE and the IS/OS.

### Retinal Thickness

Next, after mapping small pulsatile motions in the retina, we investigated the effect of anesthesia methods on a larger scale, namely on RT. In Figures 5A, 5E, and 5I, exemplary en face reflectivity images of the same male mouse at 4 time points are depicted during ISO, MMFK, and KX anesthesia, respectively. These OCT fundus images were used to manually annotate the center of the ONH. In Figures 5B, 5F, and 5J, the annular areas used to determine the RT are shown for ISO and MMFK, respectively. Higher RT can be observed at locations around the thicker blood vessels, visible in Figures 5B, 5F, and 5J. Figures 5C, 5G, and 5K show the same annular area as Figures 5B, 5F, and 5J, however, with the pixels overlapping with the SVP masked in white. These figures showcase how the SVP, which was segmented based on OCTA, encompasses more than just the major arteries and veins in the reflectivity images in Figures 5A, 5E, and 5I. RT maps for locations overlapping with the SVP vessels are shown in Figures 5D, 5H, and 5L, respectively. The RT, as shown in Figures 5B, 5F, and 5J, averaged for all mice subdivided by sex and anesthesia method is showcased in Figure 5M. Quantitative RT data derived from maps, as showcased in Figures 5C, 5G, and 5K, excluding the SVP area separated by sex and anesthetic is shown in Figure 5N. In both Figures 5M and 5N, a thicker retina, namely 206.7 and 206.0 µm, for the RT and the RT excluding the SVP (RT_-SVP_), respectively, was observed for female mice under the influence of KX, with values of 198.4 and 197.7 µm for the RT and the RT_-SVP_, respectively for female mice. The thinner RT for male mice under the influence of KX led to a statistically significant difference between male and female mice (*P* < 0.001). Moreover, the masking of the SVP influenced the RT significantly for all groups (*P* < 0.001). Figure 5O shows the RT observed at the location of the identified SVP vessels. Similarly, to Figure 5M and Figure 5N, the male mice in Figure 5O had a significantly thinner RT for KX, namely 201.0 µm compared to 209.0 µm in the female mice imaged with KX. Moreover, the mean value for the RT at the location of the SVP vessels was, on average, over all mice 2.7 µm higher compared to the RT_-SVP_, which also led to a significant difference for all groups (*P* < 0.001). In Supplementary Figure S2, spaghetti plots are shown of the RT, RT_-SVP_, and the RT_@SVP_ over time.

### Vasodilatory Analysis

In order to investigate the impact of including or excluding vascularized regions from RT measurements, the difference of the observed RT computed as ΔRT = RT - RT_-SVP_, where RT_-SVP_ is the RT where the SVP is masked. Figures 6A to 6C compare the agreement of ΔRT measured for the three anesthetics in Bland-Altman plots. On the x-axis, the mean of the ΔRT for two data points of the anesthetics are displayed whereas the y-axis depicts the difference between these points. In Figures 6A and 6B, the differences between ΔRT measurements under ISO is compared to MMFK and KX, respectively. Figure 6C shows a comparison of the effects of KX relative to MMFK. The red and cyan colored data points correspond to female and male mice, respectively. For ISO, ΔRT is, on average, 0.28 and 0.50 µm larger for female mice compared to MMFK and KX, respectively. For male mice, these differences amount to 0.38 and 0.42 µm. Interestingly, a slight segregation between data from male and female mice can be observed in these plots. Figure 3C reveals, on average, 0.24 µm smaller ΔRT for KX compared to MMFK for female mice, and similarly ΔRT was, on average, 0.25 µm smaller for male mice. In Figure 3, also a difference in distribution can be observed between male and female mice.

Figures 6D to 6F show Bland-Altman plots comparing the agreement of SVP density measured for the three anesthetics. In Figures 6D and 6E, ISO was compared to MMFK and KX. Here, ISO had a higher SVP density of 0.27 percentage points and 0.08 percentage points for female mice. However, for male mice, a lower SVP density of −0.90 percentage points and −0.51 percentage points compared with MMFK and KX was observed. Finally, Figure 6F shows the comparison between KX and MMFK. Here, a higher SVP density can be observed for female mice of 0.18 percentage points, whereas male mice exhibited a 0.46 p.p. lower SVP density.

## Discussion

The data presented in this work highlights the impact of anesthesia choice and sex of the imaged animals on the outcome of investigations of the retina. Among isoflurane, MMFK, and KX anesthesia used here, statistically significant different HRs and mean BPs were measured between female and male mice. Interestingly, in a recent study, female mice were found to have an increased resistance for volatile anesthetics.^37^ This study linked the sex differences in anesthetic sensitivity predominantly to testosterone, where castrated male mice and ovariectomized female mice showed similar results as healthy female mice. Therefore, this sex-dependent anesthetic sensitivity could explain the lower HR and higher BP for the female mice imaged with ISO. This paper demonstrated that acute testosterone increased the hypnotic sensitivity, whereas the removal of testosterone increased hypnotic resistance. Even though the authors only investigated the effects of isoflurane, other studies also reported sensitivities between sexes in both rodents and humans,^38^^–^^40^ suggesting that the effects seen with MMFK and KX could be attributed to a sex-dependent anesthetic sensitivity in general. In a different study, the effects of isoflurane and medetomidine, midazolam and fentanyl (MMF), and KX were tested on Wistar rats.^16^ While a direct comparison with our data obtained in mice can only be made cautiously because of the different species and the lack of ketamine in MMF, the general trends for the effects of anesthesia reported by the authors align with our observations. Specifically, Albrecht and coworkers similarly reported a decreased mean BP and elevated HR for ISO whereas MMF provoked elevated mean BP and a drop of the HR. The results for KX generally follow a similar trend as MMF but more moderately, which is in correspondence with our results. Note, however, that we were not able to obtain BP data for part of the measurements in male mice imaged with ISO (cf. Fig. 3C), potentially owing to the reported decrease of BP in male mice beyond the measurement range of the BP monitor. Because this could cause an overestimation of the BP in male mice, the showcased data might show a skewed representation of reality for the male mice anesthetized with ISO. In order to enhance interpretability of the data, it would provide valuable information to explore alternative methods for measuring the BP such as telemetry. It is also important to note that a previous anesthesia session might result in a potential carryover effect. Hence, in order to minimize bias, the mice were randomly assigned to three groups with permutated order of the anesthesia.

ISO also showed higher intraretinal motion compared with MMFK and KX in male and female mice at the location of the ILM. Higher MAV were observed in the OCT data for ISO compared with MMFK and KX. This could be a result of the increased HR of mice under the influence of ISO, resulting in higher pulsatile blood flow and tissue motions in the retina. In addition, in a recent study by Zhang et al., retinal tissue dynamics have been investigated.^41^ Using isoflurane as an anesthetic, the authors measured similar deformation characteristics adjacent to the retinal vessels, for instance, in the ganglion cell layer, attributing them to cardiorespiratory processes within the retina. Neurovascular studies have shown that ISO can act as a potent vasodilator and also reported strong pulsatile motion associated with ISO anesthesia.^42^^,^^43^ Thus, the increase in the ILM MAV observed for ISO in our data (cf. Fig. 4G) could possibly be caused by this dilation and pulsatile motion introduced by the blood vessels. Albeit pulsatile motion for the IS/OS was not distinctly different, the use of KX or MMFK might still be advantageous for studies using OCT methods relying on phase information such as OCE and ORG.

RT measurements exhibited anesthesia and sex-dependent differences. Our data in Figures 5M and 5N show RT values of approximately 200, which is in the range reported in the literature.^44^ Similarly to a study where the thickness of retinal layers was investigated,^45^ statistical testing did not reveal a sex-dependent difference in RT of our mouse groups using ISO and MMFK. Still, in Figures 5M and 5N, a pronounced difference can be observed between the mean and spread between the sexes when using KX. The RT analysis shown in Figures 6A, 6B, and 6C revealed that the vasodilatory effect of ISO is stronger compared to the mixture of vasodilators and vasoconstrictors in MMFK and KX.^21^^,^^24^^–^^26^ The ΔRT was, on average, 0.30 and 0.45 µm higher for ISO compared with MMFK and KX, respectively, indicating that the blood vessels in the SVP contributing to the RT are, on average, substantially larger in the axial direction when using ISO. In contrast, Figures 6D, 6E, and 6F showed a mixture of slightly higher SVP densities for female mice and slightly lower SVP densities for male mice for ISO anesthesia. Because ISO is a known vasodilator,^46^ this rather surprising finding could potentially be explained by the significantly higher BP in animals using MMFK and KX (cf. Fig. 3C), leading to faster blood flow and thus better visibility of capillary vessels in OCTA. Slow perfusion of microvasculature caused by low BP in animal subjects under ISO may eventually cause such subtle changes of the complex OCT signals during OCTA computation that microvessels could be misclassified as non-perfused tissue. Therefore, while thickness measurements of tissue geometry in beam direction (via RT) conform to literature of perfusion-related differences and thus appear to be caused by vasodilation/constriction only, blood vessel density measurements in the en face plane (via OCTA) are impacted by vasodilation/constriction, flow speed, and empirically determined, fixed OCTA threshold values. The SVP density comparison must thus be interpreted with additional caution when comparing studies performed with different anesthetic agents, as showcased here for ISO, MMFK, and KX.

The agreement analysis between experimental parameters obtained under ISO and MMFK, respectively, (cf. Fig. 6) revealed a heterogeneous picture, where the mean BP was well-correlated and the RT was poorly correlated. Finally, RT (with and without SVP) was virtually similar for ISO and MMFK and not correlated at all. However, one limitation of time-bracketing the data for each animal and both anesthesia methods was the relatively low amount of data points that could be incorporated in this part of the analysis. Whereas also the rather small sample size did allow for portraying the impact of ISO, MMFK, and KX on the retinal parameters investigated in this study, a larger sample size, the incorporation of different age ranges, and other common mouse models – in particular those of interest for preclinical OCT investigations – would make for a worthwhile expansion of the work presented here. For future experiments, it might also be interesting to expand the portfolio with different age groups or mouse models. Another option would be to measure the respiratory rate of the mice by OCT, albeit heavy breathing motion often produces image artifacts in OCT. Although we have demonstrated the quantitative assessment of the respiratory rate based on OCE in the mouse eye earlier,^33^ motion artifacts can drastically deteriorate OCT recordings, such that many of the data sets in the current study – which were based on volumetric scans rather than repeated B-scans as in Reference 33 – were not suited for measuring breathing frequency. Thus, the development of a more robust approach of OCT-based respiratory rate measurements would help to obtain a more complete overview of the physiology in mice under anesthesia. Last but not least, it could also be interesting to investigate how the outcome of functional imaging is affected by the choice of anesthesia, similar to what has been demonstrated in rats before but possibly expand the research with the usage of ORG.^47^ The hypothesis stated in the introduction section has mostly been confirmed by the outcome of the experiments. OCT parameters as well as the physiological parameters were markedly influenced by the choice of anesthesia. Our observations on the physiological effects of the anesthetics agreed well with previous reports from literature, although sex-dependent differences had not been exhaustively investigated yet. As for the OCT parameters describing pulsatile motion, MAV at the IS/OS did not differ considerably among the three anesthesia approaches, whereas at the ILM a statistically significant difference of MAV was observed. Our results also showed that the choice of anesthesia did affect ΔRT but not RT, likely due to the relative vasodilatory properties between the anesthetics.

## Conclusions

This work highlights the importance of standardizing research within the research community. The three anesthetics investigated here are excellent choices for structural OCT imaging in the murine retina, but temporarily change the physiology in different distinct manners. In order to create a translational research environment, it is imperative to have research which can be compared to each other.

## Supplementary Material

Supplement 1
