# Supplementary material for: Retinal OCT Parameters and Physiological Dynamics as an Effect of Anesthesia in C57BL/6J Mice
Source: Invest Ophthalmol Vis Sci. 2026 Apr 21;67(4):50. doi: 10.1167/iovs.67.4.50 (PMC13104790; doi:10.1167/iovs.67.4.50)
Supplement: Supplement 1 [file iovs-67-4-50_s001.pdf]

Supplementary information

Variable	contrast	Additional info	β	p value	CI	Cohen_d	Power
HR	ISO - KX	M	207.78	1.79E-10	64.16	2.80	1.00
HR	ISO - MMFK	M	107.05	1.12E-03	70.34	1.44	0.77
HR	KX - MMFK	M	-100.7	1.44E-03	67.60	-1.36	0.77
HR	ISO - KX	F	171.90	1.36E-08	61.36	2.32	1.00
HR	ISO - MMFK	F	122.51	3.21E-05	62.56	1.65	0.87
HR	KX - MMFK	F	-49.38	0.20	65.17	-0.67	0.24
HR	M - F	ISO	47.53	0.07	51.11	0.64	0.22
HR	M - F	KX	11.65	0.65	50.83	0.16	0.06
HR	M - F	MMFK	62.99	0.03	56.92	0.85	0.35
BP	ISO - KX	M	-30.671	0.018	26.530	-2.462	0.825
BP	ISO - MMFK	M	-27.892	2.7e-4	16.897	-2.239	0.752
BP	KX - MMFK	M	2.779	1.000	25.112	0.223	0.070
BP	ISO - KX	F	-17.234	0.346	26.624	-1.384	0.730
BP	ISO - MMFK	F	-22.906	0.107	26.297	-1.839	0.927
BP	KX - MMFK	F	-5.672	1.000	27.354	-0.455	0.136
BP	M - F	ISO	-18.212	0.097	21.591	-1.462	0.413
BP	M - F	KX	-4.775	0.659	21.576	-0.383	0.110
BP	M - F	MMFK	-13.226	0.213	21.035	-1.062	0.507
MAV ILM	ISO - KX	M	1.39	3.05E-05	0.71	2.99	1.00
MAV ILM	ISO - MMFK	M	0.86	0.019	0.75	1.85	0.93
MAV ILM	KX - MMFK	M	-0.53	0.22	0.72	-1.14	0.62
MAV ILM	ISO - KX	F	1.70	4.82E-07	0.71	3.67	1.00
MAV ILM	ISO - MMFK	F	1.55	3.64E-06	0.70	3.33	1.00
MAV ILM	KX - MMFK	F	-0.16	1	0.75	-0.34	0.10
MAV ILM	M - F	ISO	0.65	0.025	0.57	1.41	0.74
MAV ILM	M - F	KX	0.97	1.50E-03	0.58	2.08	0.97
MAV ILM	M - F	MMFK	1.34	4.33E-05	0.61	2.88	1.00
MAV ISOS	ISO - KX	M	0.53	0.43	0.88	1.36	0.72
MAV ISOS	ISO - MMFK	M	0.55	0.43	0.91	1.42	0.75
MAV ISOS	KX - MMFK	M	0.02	1.00	0.87	0.05	0.05
MAV ISOS	ISO - KX	F	0.93	0.03	0.87	2.39	0.99
MAV ISOS	ISO - MMFK	F	0.74	0.13	0.87	1.89	0.94
MAV ISOS	KX - MMFK	F	-0.19	1.00	0.93	-0.50	0.15
MAV ISOS	M - F	ISO	0.38	0.28	0.71	0.99	0.45
MAV ISOS	M - F	KX	0.78	0.03	0.72	2.02	0.96

MAV ISOS	M - F	MMFK	0.57	0.13	0.74	1.47	0.78
RT	ISO - KX	M	1.87	1.00	5.50	0.56	0.18
RT	ISO - MMFK	M	-2.86	0.68	5.78	-0.85	0.35
RT	KX - MMFK	M	-4.74	0.11	5.49	-1.40	0.80
RT	ISO - KX	F	-3.53	0.32	5.32	-1.05	0.55
RT	ISO - MMFK	F	-3.53	0.35	5.45	-1.05	0.50
RT	KX - MMFK	F	0.00	1.00	5.63	0.00	0.05
RT	M - F	ISO	-3.21	0.15	4.44	-0.95	0.43
RT	M - F	KX	-8.62	1.96E-04	4.35	-2.55	1.00
RT	M - F	MMFK	-3.88	0.10	4.68	-1.15	0.57
RT -SVP	ISO - KX	M	1.82	1.00	5.46	0.54	0.17
RT -SVP	ISO - MMFK	M	-2.88	0.67	5.74	-0.86	0.36
RT -SVP	KX - MMFK	M	-4.70	0.11	5.45	-1.40	0.80
RT -SVP	ISO - KX	F	-3.71	0.27	5.28	-1.11	0.60
RT -SVP	ISO - MMFK	F	-3.59	0.32	5.41	-1.07	0.52
RT -SVP	KX - MMFK	F	0.12	1.00	5.59	0.04	0.05
RT -SVP	M - F	ISO	-3.10	0.16	4.41	-0.93	0.41
RT -SVP	M - F	KX	-8.63	1.75E-04	4.32	-2.58	1.00
RT -SVP	M - F	MMFK	-3.81	0.11	4.65	-1.14	0.56
RT@SVP	ISO - KX	M	2.45	0.92	-3.41	0.69	0.25
RT@SVP	ISO - MMFK	M	-2.86	0.80	-9.15	-0.81	0.33
RT@SVP	KX - MMFK	M	-5.32	0.10	-11.29	-1.50	0.80
RT@SVP	ISO - KX	F	-2.62	0.78	-8.32	-0.74	0.32
RT@SVP	ISO - MMFK	F	-3.45	0.45	-9.28	-0.98	0.44
RT@SVP	KX - MMFK	F	-0.83	1.00	-6.86	-0.23	0.07
RT@SVP	M - F	ISO	-3.45	0.15	-8.19	-0.98	0.44
RT@SVP	M - F	KX	-8.52	5.15E-04	-13.17	-2.41	1.00
RT@SVP	M - F	MMFK	-4.04	0.12	-9.14	-1.14	0.57

Supplementary Table 1. Statistical information. BP blood pressure; CI confidence interval; F female; KX ketamine-xylazine; HR heart rate; ISO isoflurane; M male; MAV mean absolute velocity for the ISOS and the ILM; MMFK medetomidine; midazolam; fentanyl and ketamine; RT; RT excluding areas at SVP; and the RT solely at the locations of the SVP.

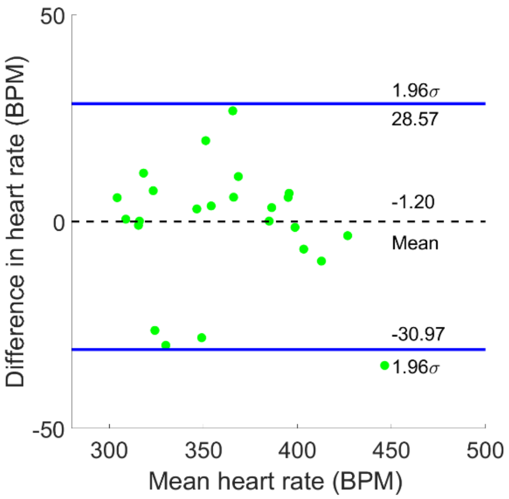

Supplementary Figure 1. Bland-Altman plot showing the agreement of two different methodologies to determine heart rate. Method 1 was based on the heart rate assessed with a tail cuff-based blood pressure monitor, whereas method 2 was based on an algorithm analysing the OCT data.

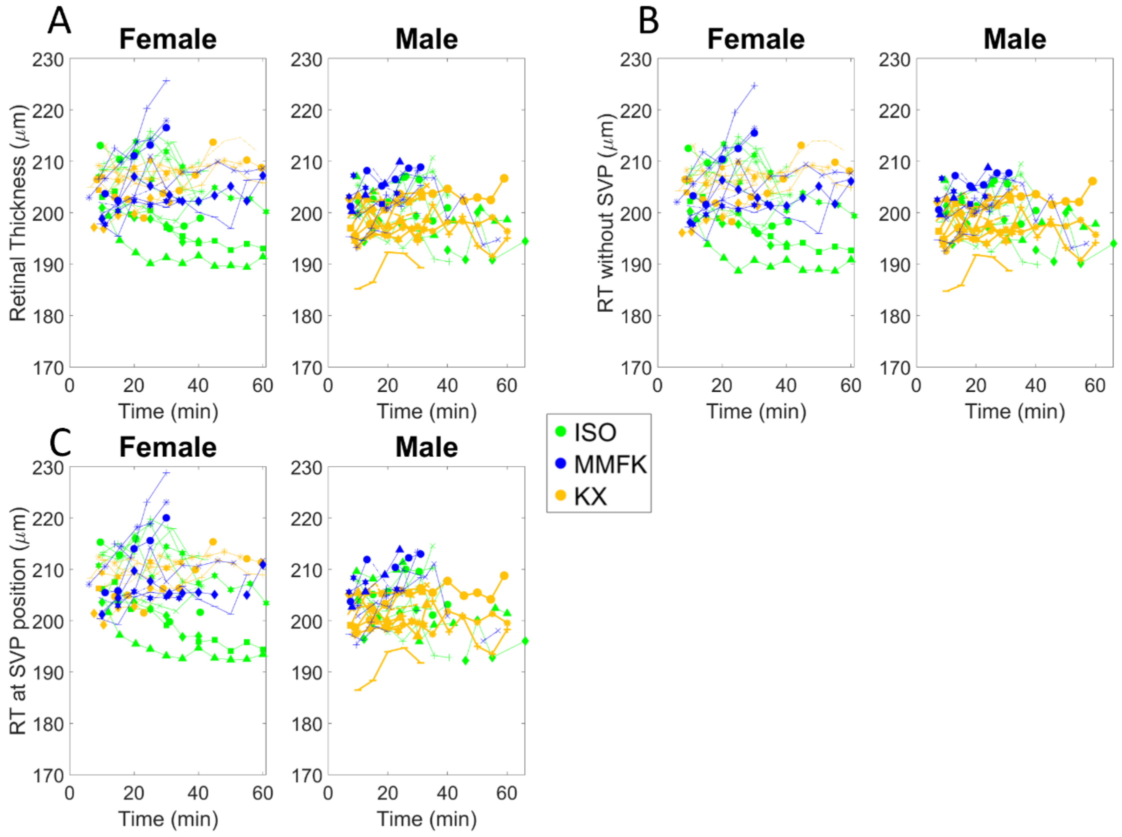

Supplementary Figure 2. Longitudinal retinal thickness measurements. Each spaghetti plot represents the retinal thickness data for an individual mouse over time: (A) Retinal thickness. (B) Retinal thickness including locations coinciding with SVP vasculature. (C) Retinal thickness at locations coinciding with SVP vasculature.

	**Heart Rate**		**Blood Pressure**		**MAV ILM**		**MAV ISOS**	
**Effect**	**F**	**p**	**F**	**p**	**F**	**p**	**F**	**p**
Anesthetic	14.69	1.1E-06	31.49	9.3E-12	26.43	6.4E-10	7.77	1.0E-03
Sex	0.09	0.76	10.30	1.8E-3	20.46	1.6E-5	9.65	2.9E-03
Time	28.11	1.9E-07	0.10	0.74	75.18	2.3E-16	25.08	9.7E-07
Anesthetic x Sex	0.03	0.97	0.89	0.41	0.27	0.76	0.45	0.64
Anesthetic x Time	4.07	0.02	21.65	2.2E-09	3.35	0.04	4.85	8.5E-03
Sex x Time	3.84	0.05	8.36	4.2E-3	0.93	0.34	0.63	0.42
Anesthetic x Sex x Time	0.52	0.60	0.74	0.48	1.12	0.33	2.19	0.11

	**RT**		**RT - SVP**		**RT @ SVP**	
**Effect**	**F**	**p**	**F**	**p**	**F**	**p**
Anesthetic	4.63	0.01	4.27	0.02	5.92	3.7E-3
Sex	9.91	2.2E-3	9.64	2.4E-3	8.70	4.0E-3
Time	1.35	0.25	1.34	0.25	1.29	0.26
Anesthetic x Sex	2.14	0.12	2.25	0.11	1.16	0.32
Anesthetic x Time	29.09	2.2E-12	28.12	5.1E-12	29.45	2.0E-12
Sex x Time	0.87	0.35	0.96	0.33	0.82	0.37
Anesthetic x Sex x Time	5.81	3.3E-3	6.09	2.5E-3	3.17	0.04

Supplementary Table 2. Type 3 analysis of variance table showing both the F value and the corresponding p values for each effect combination and primary outcome.
